# Genetic Association Between *CD143* rs4340 Polymorphism and Pneumonia risk: A Meta Analysis

**DOI:** 10.1097/MD.0000000000000883

**Published:** 2015-07-31

**Authors:** Hong Wang, Kun Zhang, Haifeng Qin, Lin Yang, Liyu Zhang, Yanyan Cao

**Affiliations:** Department of Lung Cancer, 307 Hospital of PLA, Affiliated Hospital of Academy of Military Medical Sciences, Beijing, China.

## Abstract

rs4340 polymorphism at intron 16 of the angiotensin-converting enzyme (*CD143*) gene was reported to repress cough reflex by reducing bradykinin and substance P levels, thus increasing the likelihood to develop pneumonia. There have been different reports regarding the correlation of *CD143* rs4340 genotypes with pneumonia risk, which prompted us to perform a meta-analysis to determine the elusive association.

We combined multiple keywords to identify the studies addressing the association between *CD143* rs4340 genotypes and pneumonia risk covered in the EMBASE, Google Scholar, PubMed, and CNKI databases. Odds ratios (ORs) and 95% confidence intervals (CIs) were calculated to assess the risk of pneumonia. The fixed-effects model (FEM) was used.

A total of 10 studies were analyzed in this quantitative analysis. We found a strong association between rs4340 single nucleotide polymorphism (SNP) and pneumonia risk using the recessive model (FEM: OR 1.33, 95% CI 1.13–1.57). A significantly increased risk was also indicated under the recessive model in Asian populations (FEM: OR 1.57, 95% CI 1.19–2.07), community-acquired pneumonia (CAP) (FEM: OR 1.31, 95% CI 1.08–1.60), and nosocomial pneumonia (NP) (FEM: OR 1.52, 95% CI 1.06–2.19).

Our meta-analysis demonstrates that *CD143* rs4340 polymorphism may represent a risk factor for pneumonia.

## INTRODUCTION

Pneumonia stemming from virus or bacteria infections of the alveoli is a frequent respiratory disease that has caused quite a large number of deaths worldwide.^1^ Much research has been done to approach the etiological factors, with especially extensive attention given to the renin-angiotensin system due to the potential involvement in initiation and progression of pneumonia.

Available data implicate that pathogenesis of pneumonia correlates with attenuation of cough reflex,^2,3^ an important defensive reflex in human body mediated by substance P and an inflammatory peptide bradykinin through evoking sensitization of airway sensory nerves.^4,5^ A reduced concentration of substance P has been reported in sputum of patients with pneumonia.^6^ These findings make many groups hypothesize that the enzymes involved in the regulation of substance P and bradykinin may possibly have a causal role in pneumonia. Angiotensin-converting enzyme (*CD143*) is such a regulatory enzyme essential for the conversion of angiotensin-I (AT-I) to AT-II, a product in the process of proteolytic cleavage of angiotensinogen.^7^*CD143* inhibitors have been shown to decrease the risk to develop pneumonia in elderly individuals and a reduction in mortality rate has been reported in patients who use *CD143* inhibitors.^8,9^ These data indicated that a clearer understanding of the correlation between *CD143* and pneumonia will help prevent or survive this disease.

The human *CD143* gene on chromosome 17q23 spans over 24 kb in length and contains 26 exons. Serum *CD143* levels are genetically determined and around half of the variance arises as a result of a functionally important insertion/deletion (I/D, presence/absence) polymorphism (rs4340) at intron 16. An association of the D-allele with enhanced *CD143* activity has previously been established.^10^ The same allele has been linked with a wide range of diseases, ranging from cardiovascular events and renal disorders.^11^ Moreover, individuals harboring the D-allele are shown to have greater risk of pneumonia.^12^ The I/I genotype, however, contributes to a high chance of survival for patients with acute respiratory distress syndrome.^13,14^

Published epidemiological studies reported different conclusions regarding the role of rs4340 single nucleotide polymorphism (SNP) in pneumonia predisposition.^15,16^ We therefore performed a meta-analysis, attempting to clarify whether or not the rs4340 SNP is correlated with risk of pneumonia by means of meta-analysis.

## METHODS

### Ethics Statement

This study was approved by the PLA 307th Hospital Ethics Committee. This study does not involve patients, so ethical approval was not required.

### Publication Search Strategy

We combined “angiotensin-converting enzyme,” “*ACE*,” “*CD143*,” “polymorphism,” “genotypes” “variants,” “pneumonia,” “community-acquired pneumonia,” and “acute respiratory distress syndrome” to identify the case-control studies covered in the EMBASE, Google Scholar, PubMed, and CNKI databases. All research articles addressing the correlation of *CD143* rs4340 SNP with pneumonia risk were checked for their references to obtain additional data. We also manually searched the journals known to publish pneumonia-related articles. The last search was undertaken in October 2013.

### Selection Criteria

Eligible studies were selected based on: pneumonia patients were investigated; reference group consisted of healthy individuals; the association of *CD143* rs4340 SNP with pneumonia risk was evaluated; genotype distribution must be clearly presented; the case group consisted of at least 30 patients. Any study that violated the aforementioned requirements was certainly excluded from this analysis. When more than 1 study employed the same cases, we selected the study with the largest number of subjects.

### Data Extraction

Two investigators strictly followed the inclusion criteria to select all eligible studies. Data extraction was independently performed. Information on authors’ surname name and institute, publication year, study country, racial descent (ethnicity), source of controls, determination of rs4340 polymorphism, type of pneumonia, number of cases and controls, and genotype frequency was collected for each study. Racial descent was grouped as Caucasian, Asian, or mixed when ethnic origin was unclear; type of pneumonia was classified as community-acquired pneumonia (CAP), nosocomial pneumonia (NP), or information inaccessible (II) when data were not stated. Disagreements were resolved through discussion among all investigators and a final decision was based on major opinions.

### Statistical Methods

Both overall and stratified meta-analyses (by ethnicity and type of pneumonia) were performed in this study. Using the allele and genotype data, we assessed the risk of pneumonia (OR, odds ratio) with an assumption of D/D versus I/I (homozygote model), I/D versus I/I (heterozygote model), D/D + I/D versus I/I (dominant model) and D/D versus I/D + I/I (recessive model).

Between-study heterogeneity was evaluated by Cochran Q statistic,^17^ and *P* values less than 0.05 were considered significant. In such a case, the random-effects model (REM) derived from the DerSimonian–Laird method was used to summarize the values for each study,^18^ or else the fixed-effects model (FEM, Mantel–Haenszel method) was applied.^19^ Begg funnel plots and Egger linear regression test were utilized to determine publication bias.^20,21^ Sensitivity analysis was performed to check if the single studies had significant influence on the overall meta-analysis results. The genotype distribution in control groups was checked for Hardy–Weinberg equilibrium (HWE) deviation.

All statistical analyses were done using the STATA version 12.0 (Stata Corporation, College Station, TX). Two-tailed *P* values were considered significant when *P* < 0.05.

## RESULTS

### Study Inclusion and Characteristics

**FIGURE 1:**
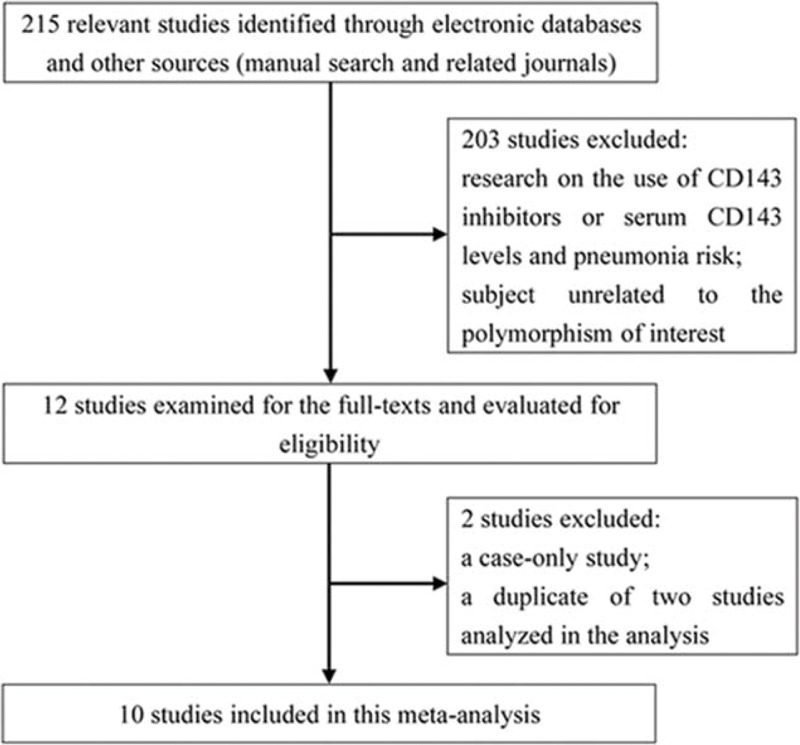
The flow chart of the included studies for a meta-analysis of *CD143* rs4340 polymorphism and pneumonia risk.

As depicted in Figure 1, 215 potentially relevant studies were identified through electronic databases and other sources. Among these, 203 studies were excluded owing to research concerning use of *CD143* inhibitors or serum *CD143* levels and pneumonia risk, or subjects unrelated to the polymorphism of interest. After reading the full texts of the remaining articles, we further excluded 2 studies, of which 1 was a case-only study,^22^ and the other was a duplicate of 2 studies analyzed in the present analysis.^23^ Therefore, 10 original articles concerning the correlation of rs4340 SNP with pneumonia were considered eligible for this analysis.^15,16,24–31^ Main characteristics, including first author, publication year, study country, ethnicity of the populations included, assays used in determining rs4340 SNP, source of controls, specific type of pneumonia, and sample size are presented in Table 1. The 10 studies from 6 different countries were composed of 4 Caucasian studies, 5 Asian studies, and 1 study without stating the ethnicity. In addition, 7 groups studied CAP, 2 studied NP (Takahashi et al presented data in forms of D/D, I/D + I/I, so this study was only available for the recessive model), and 1 study did not give any information on which type of pneumonia was studied. All genotype frequencies were in HWE with the exception of Gu et al (*P* < 0.001).

**TABLE 1:**
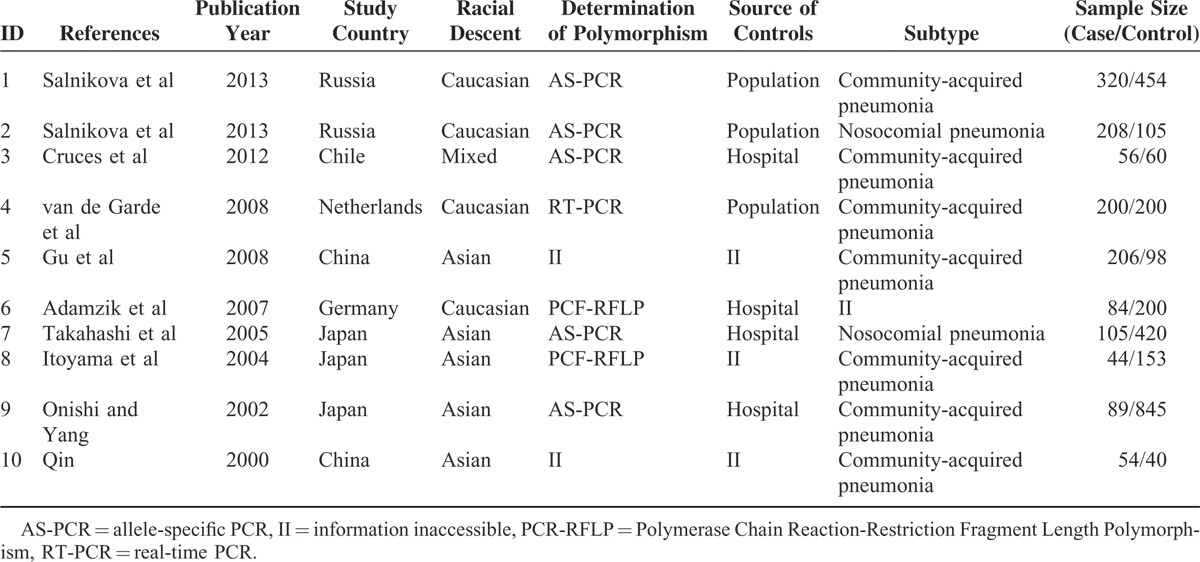
Baseline Characteristics of the Studies Considered in the Meta-Analysis

### Quantitative Synthesis

**FIGURE 2:**
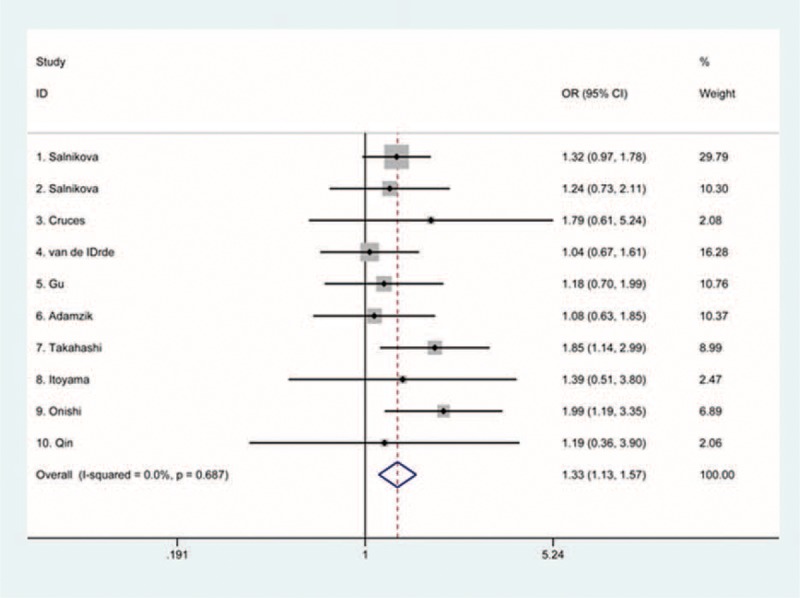
Meta-analyses for functional rs4340 polymorphism in pneumonia risk under the recessive model. CI = confidence interval, OR = odds ratio.

We performed a meta-analysis among 1366 cases and 2575 controls in this quantitative analysis, as shown in Table 2. A strong association between rs4340 SNP and pneumonia risk was indicated using the recessive model (FEM: OR 1.33, 95% confidence interval [CI] 1.13–1.57) (Figure 2). The homozygote model showed a relatively weaker association with risk of pneumonia (FEM: OR 1.20, 95% CI 1.00–1.45).

**TABLE 2:**
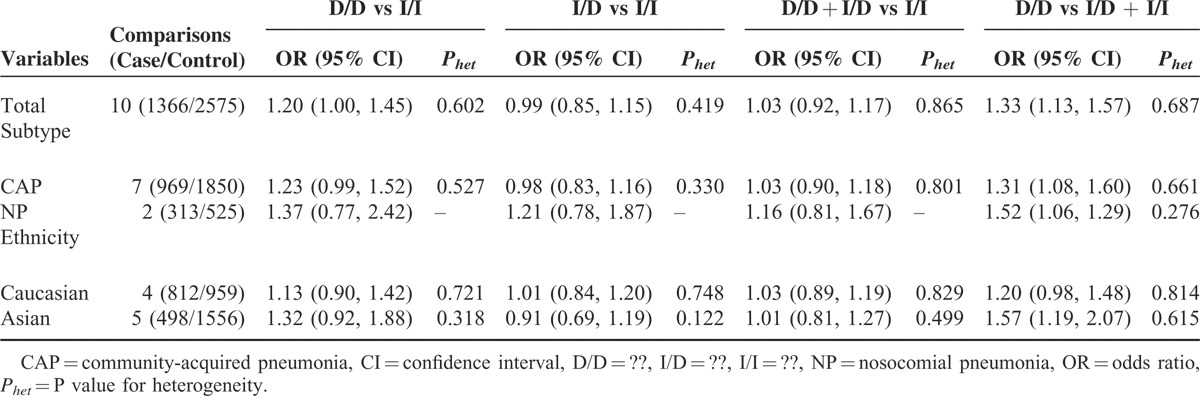
Summary ORs for the Association of *CD143* rs4340 SNP With Pneumonia Risk

In the stratified analysis according to ethnicity, we found a significantly increased pneumonia risk under the recessive model in Asian populations (FEM: OR 1.57, 95% CI 1.19–2.07). Stratification analysis by type of pneumonia showed that rs4340 was associated with notably increased risk of both CAP (FEM: OR 1.31, 95% CI 1.08–1.60, the recessive model) and NP (FEM: OR 1.52, 95% CI 1.06–2.19, the recessive model).

### Sensitivity Analysis

The independent studies, including the Chinese study with HWE exclusion, were sequentially excluded to examine if the recalculated ORs, compared to the primary combined effects, were significantly different. We found no notable changes throughout the analysis, suggesting our results are stable (figure not shown).

### Publication Bias

**FIGURE 3:**
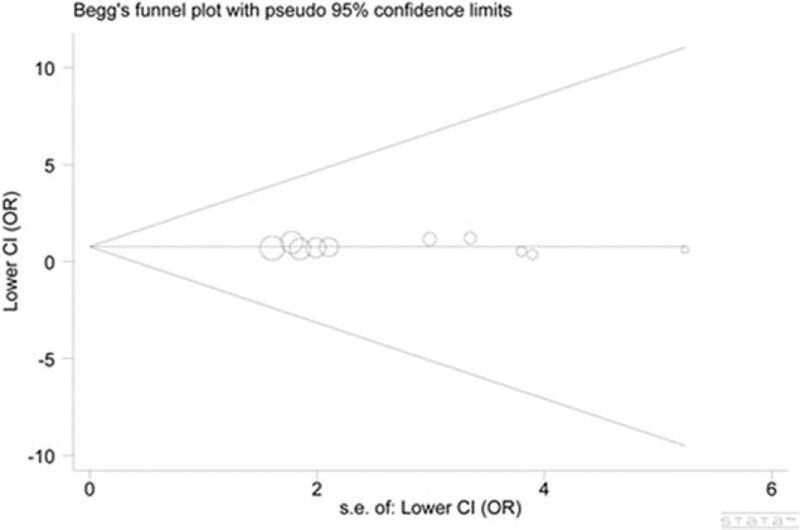
Funnel plots of the Egger test of *CD143* rs4340 polymorphism for publication bias. CI =  confidence interval, OR = odds ratio.

In the funnel plots for each genetic model, the studies were symmetrically distributed. When performing the Egger test, we found little evidence of significant publication bias across studies (Begg, *P* = 0.721; Egger, *P* = 0.975, the recessive model) (Figure 3).

## DISCUSSION

Multiple lines of work have consistently correlated rs4340 in the *CD143* gene with pneumonia risk in the past decade. The previous conflicting reports, however, fail to provide compelling evidence, thereby making the association between rs4340 SNP and pneumonia risk elusive. For instance, a prospective case-control study demonstrated data that did not support a causal association of rs4340 genotypes with pediatric pneumonia.^16^ A most recent study in a Russian population suggested that rs4340 genotypes accounted for the increase in vulnerability to CAP and NP.^15^ The discrepancy may result from different study designs and distinct ethnic groups. Other possible factors such as the number of subjects analyzed and differences in clinical features and methodology may also cause the inconsistency in results.

We performed a meta-analysis in this study, attempting to determine the association between rs4340 polymorphism and pneumonia risk. We analyzed a total of 3941 subjects from 10 case-control studies. When all data were combined, we found rs4340 D/D genotype was associated with 1.33-fold increased risk of pneumonia. An obviously higher risk was observed in Asian samples in stratified analysis by racial descent. We also noted that individuals with the same genotype had notably greater risk of CAP and NP, which is consistent with the study by Salnikova et al. ^15^ However, both CAP and NP are etiologically heterogeneous and involvement of confounders such as gender, diabetes mellitus, duration of surgery, mechanical ventilation, obesity, and urinary tract catheterization may further complicate the etiology of these diseases.^32,33^ Therefore, the exact role of rs4340 genotypes play in pneumonia requires further validation.

A recently published meta-analysis concerning the same topic suggested a significant association with pneumonia risk.^34^ In this work, the authors combined 1431 cases and 3600 controls, of which 977 samples should be excluded (the reason was described in study inclusion and characteristics). In addition, the data included were not all in accord with those provided in original articles. It is the above-mentioned reasons along with the current discrepancy in results that prompted us to perform a meta-analysis. We noted that the association indicated in the previous analysis was relatively stronger compared to this study, suggesting rs4340 genotypes may have substantial effects on pneumonia risk.

The findings in our study are supported by accumulated functional evidence. Brown et al provided evidence that rs4340 genotypes induce cough by suppressing the tissue levels of bradykinin and substance P; the I/I and I/D genotypes had a higher cough reflex relative to the D/D genotype.^35,36^ D/D genotype was also reported to increase serum levels of the proinflammatory AT-II.^37^ It is known that reduced bradykinin and substance P levels repress cough reflex and therefore increase the risk to develop pneumonia. Based on these data, we infer that the D/D genotype of rs4340 polymorphism is associated with increased risk of pneumonia, an inference in line with the positive associations revealed in the present study.

Our study shares the same limitations with most meta-analyses. First, publication bias arises due to many reasons and 1 of them relates to inclusion of small studies. We included several studies with a small number of subjects, leading to possible publication bias that often affects the precision of overall results. Second, there is statistically significant difference in D allele frequency between Caucasians and Asians (53.14% vs 38.42%, *P* < 0.05). Due to the significantly higher allele frequency, Caucasians are theoretically more susceptible to pneumonia than Asians, which is inconsistent with the current findings. Third, deviation from HWE probably caused by methodological errors may affect the results in original article and consequently biases the meta-analysis results.

In conclusion, this meta-analysis shows evidence supporting a causal association between rs4340 SNP in the *CD143* gene and pneumonia vulnerability. A similar trend was indicated in subgroups of Asian populations, CAP and NP. Future studies are urgently needed to address the pathogenic role that remains poorly understood.
